# Measurement and modeling of temperature evolution during methane desorption in coal

**DOI:** 10.1038/s41598-020-59589-w

**Published:** 2020-02-21

**Authors:** Yue Gaowei, Zeng Chunlin, Huo Liupeng, Zheng Xinjun

**Affiliations:** School of Civil Engineering, Henan Polytechnic University, Jiaozuo, Henan 454000 China

**Keywords:** Natural hazards, Coal

## Abstract

The decrease of coal temperature has been confirmed by many tests during methane desorption in coal, including coal and gas outburst, but the thermal-dynamic process for methane desorption has not been quantitatively studied. Therefore, firstly, the coal temperature and gas pressure are measured by temperature and pressure sensors in the process of methane desorption. Secondly, isosteric heats of adsorption are calculated according to the adsorption isotherm. Finally, heat transfer model is established and simulate the temperature evolution during methane desorption in coal under different conditions (initial temperature and gas pressure). The real tests and simulation results show that a lot of heat will be absorbed from coal as methane desorbing, which causes the coal temperature will go down by 5.5 K, and methane desorption is no longer isothermal process. In the initial stage of methane desorption in coal, the coal temperature will decrease sharply to an extremely low value, then slowly rise to the previous ambient temperature. And at the same ambient temperature, the higher the initial methane equilibrium pressure is, the larger the temperature at the coal body center drops in the process of methane desorption. In the coal body, the farther away from the wall of the coal sample canister, the more significant the decrease of the coal body temperature is, and the longer the time is to reach extremely low value, which is mainly due to the different heat transfer coefficients at different positions in the coal body. The total specific power, which is a key index in heat transfer model to simulate the change of coal temperature, sharply decreases during methane desorption, because the methane desorption quantity in unit time decreases gradually. This study has an important practical significance to reveal the evolution mechanism of coal and gas outburst, and predict outburst with temperature change as an index.

## Introduction

Coal and gas outburst is one of the most serious disasters in coal mine production, and the outburst process is often accompanied with abnormal temperature. Practice results show that: Before coal and gas outburst, the coal wall becomes cooler, and the air temperature decreases in the working face^1^. This is because gas desorption is an endothermic process, and a large amount of heat is absorbed from coal body due to gas rapid desorption during the outburst process, which results lower temperature of coal body^2–4^. The laws of coal temperature reduction, induced by methane absorbing heat from coal to desorb, not only can objectively evaluate the methane desorption characteristics in coal, but also can further understand the evolution mechanism of coal and gas outburst, which has great significance for coal mine safety production^5–7^.

In recent years in unconventional natural gas development fields, more and more researchers begin to pay attention to the thermal-dynamic process of gas ad/desorption in porous media^4,8^. In the working face of coal mining, the phenomenon of coal temperature reduction has been observed and confirmed before coal and gas outburst, therefore, the sudden change of coal temperature can be used as an index to predict coal and gas outburst disasters^9^. Many experimental tests have been carried out to analyze the laws of coal temperature change in the process of methane desorption^9,10^. Although different techniques have been used to describe the thermal effect induced by the heat of desorption during methane desorption process, the thermal-dynamic mechanism of methane desorption is still unclear, the heat of desorption affected by initial pressure and temperature in coal has not yet been seriously considered, even this influence is always neglected^11–13^. So the thermal-dynamic model during methane desorption is necessary to quantitatively study temperature change of coal body under different environmental temperature and pressure. Furthermore, previous experiments of methane desorption in coal are carried out above or room temperature, and the temperature below 0 °C has rarely been considered.

In order to explore the evolution law of coal body temperature and the thermal effect induced by the heat of desorption in the process of methane desorption in coal, the coal temperature and gas pressure in coal sample canister are measured by temperature and pressure sensors *via* in-house adsorption and desorption test system with temperature control from −50 °C to 100 °C^13,14^. Then, the heat transfer model of methane desorption is established to simulate the change laws of coal temperature, which can reveal the thermal-dynamic mechanism as an index to predict coal and gas outburst.

## Gas Desorption Test System and Test Methodology

### Sample preparation

The blocks of coking coal were taken from the Xingwu coal mine, Lvliang, Shanxi province of China. The physical parameters of the coal were evaluated using Chinese national standards (GB/T 212-2008) shown as follows: ash content (*A*_*ad*_) 19.4%, volatile matter (*V*_*ad*_) 6.66%, moisture (*M*_*ad*_) 2.64%. The coal specimen was then ground and sieved with 0.17−0.25 mm metal sifters.

**Figure 1:**
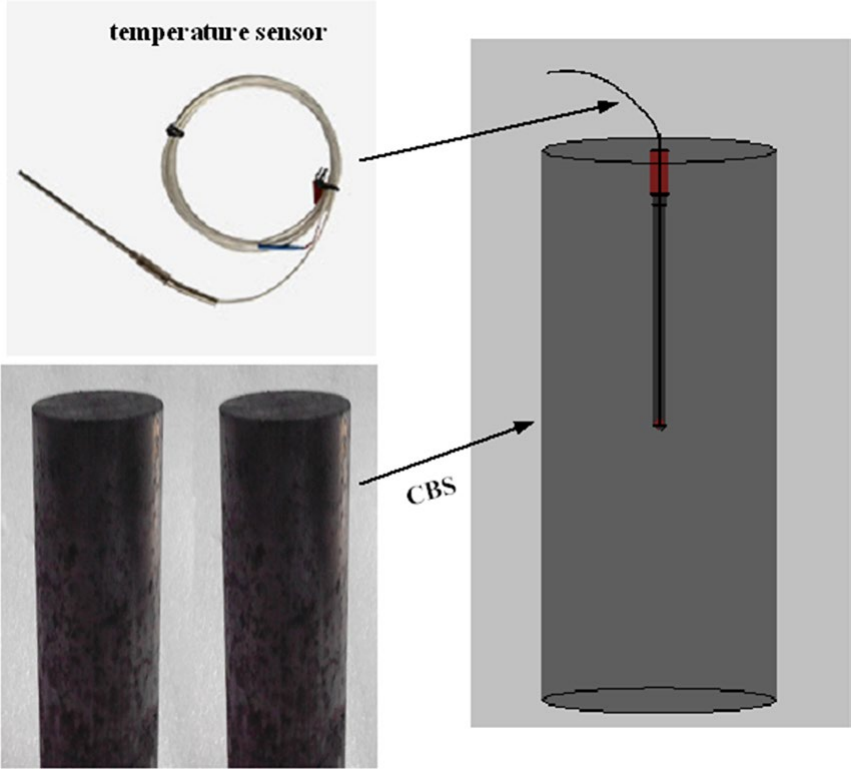
Coal briquette specimen.

A manufacturing process of coal briquette specimen (CBS) is introduced as follows: (1) The amount of distilled water is added to 0.17 mm~0.25 mm coal specimen and then stired evenly; then (2) the granular coal is placed into the mold and pressed under the pressure of 60 kN using RMT-150B type electro-hydraulic servo testing machine; (3) the CBS is obtained after demoulding, as shown in Fig. 1; (4) the CBS is placed into a drying oven at 377.15−383.15 K for 24 h to dehydrate; (5) From one end of CBS, a borehole of 2.5 mm diameter along axis direction is drilled carefully to the middle position, and the temperature sensor is inserted into the borehole and sealed, as shown in Fig. 1. Then the prepared sample was stored in a dehydrator for later use (GB/T 212-2008).

### Test system

**Figure 2:**
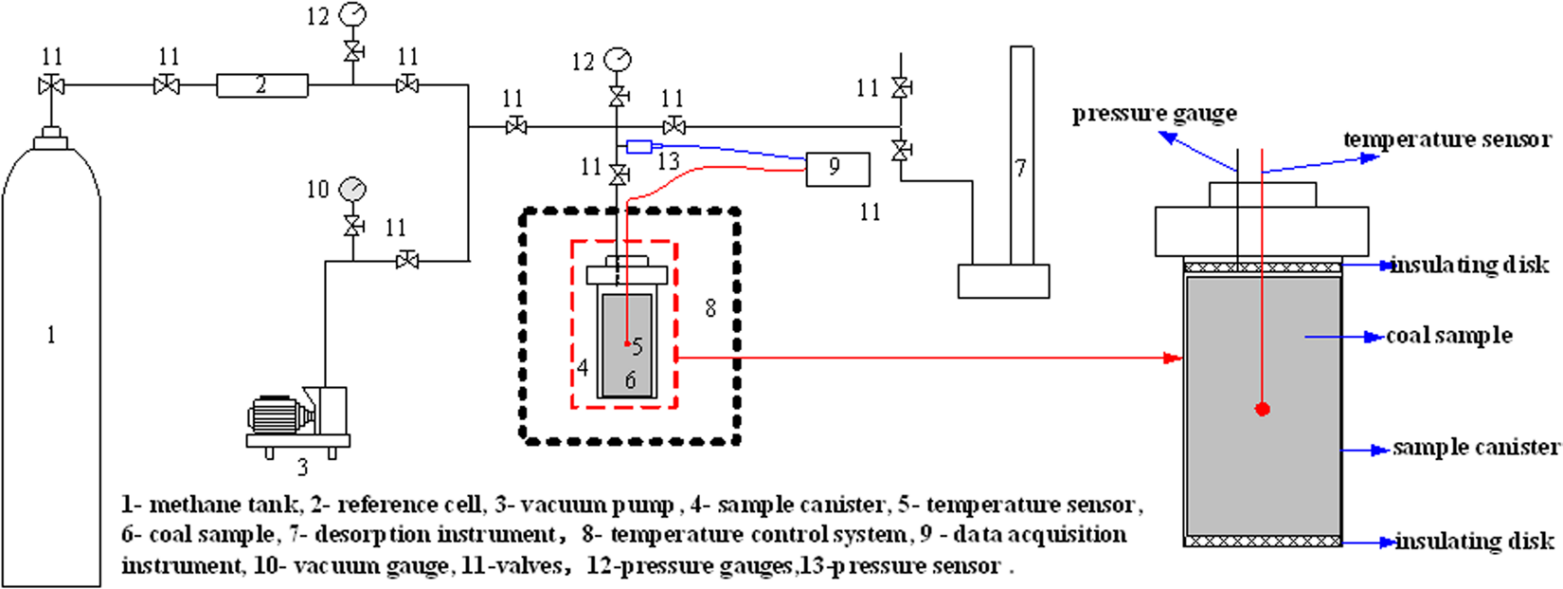
Schematic setup for temperature measurement during the methane desorption process in coal.

**Figure 3:**
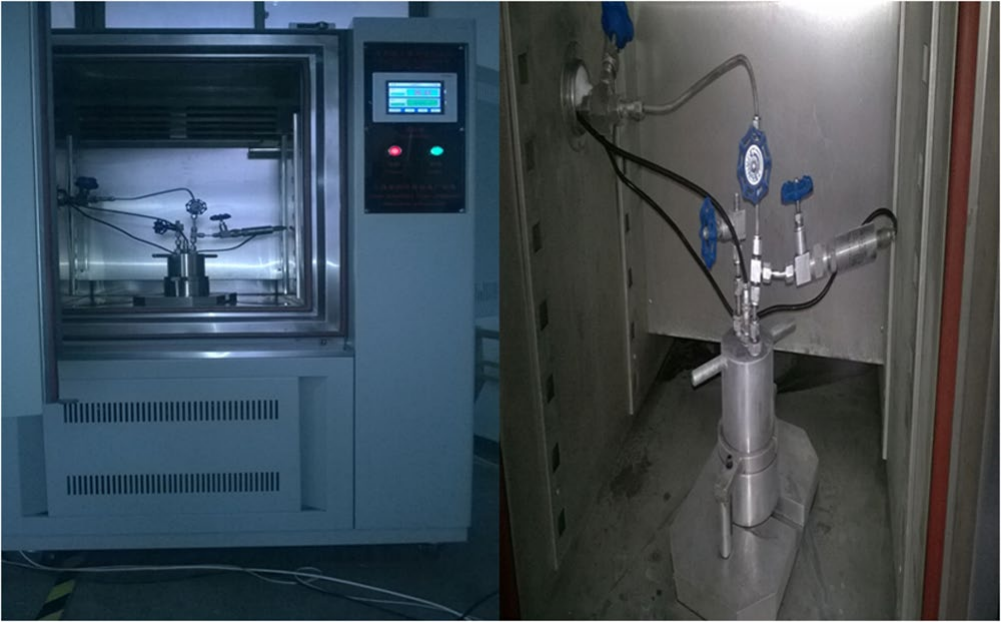
Control system for High/low temperature and multifunctional coal tank.

Figure 2 shows the experimental device for gas desorption and temperature test in coal, which can control a large range of test temperature from −50 °C to 100 °C by temperature frequency conversion box (shown as Fig. 3), and the temperature deviation is less than 1 °C and the temperature fluctuation is less than 0.5 °C.

The test process of gas desorption in coal at 273.15 K is taken as an example to show the coal temperature measurement procedure. And it should be noted that the temperature sensor is placed in the geometric center of the coal sample inside the canister in each test.

1. Calibrate the sample cell volume and double-check the tightness of the whole test system.
2. Place the prepared coal sample into the coal canister, and put the canister into the temperature control system (shown in Fig. 3).
3. Set the temperature control system (in Fig. 2) to 273.15 K, and vacuum the sample canister to at least 10 Pa. At the same time, measure the temperature of the coal using the temperature gauge until the temperature of coal reaches 273.15 K.
4. Once the temperature of the coal inside the canister remains at 273.15 K, methane will be filled into the canister *via* the reference gas cell, and the pressure of the canister will be monitored and recorded simultaneously.
5. When the coal temperature and the gas pressure in the canister remain unchanged for 2 h, methane desorption is conducted, and the temperature of the coal and the amount of gas desorption can be monitored and recorded simultaneously.
6. Repeat steps 1−5 until all the scheduled tests under different temperatures are conducted.

### Test plan

In this test, temperature evolution induced by the heat of desorption will be monitored and investigated during methane desorption in coal. First, the center temperature of the coal sample inside the canister will be measured in the following methane desorption process. The reason why the center is measured is that the temperature variation of the sample center will be the most pronounced case. Then, the desorption experiments are conducted to obtain isothermal desorption curves, which is used to calculate the isosteric heats of adsorption in desorption process. Next, the heat transfer model will be developed. The temperature evolution in the center of the coal sample will be simulated and validated by experimental data first. Finally, the temperature of the coal at different places inside the canister will be modeled.

## Test Results and Analysis

### Reliability of test results

**Figure 4:**
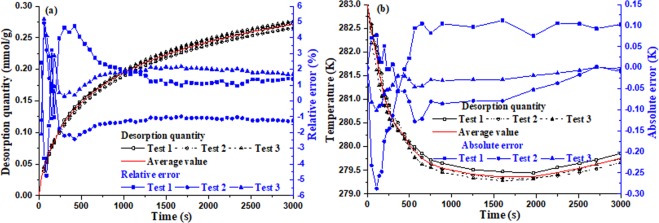
Error analysis of the results on desorption quantity and coal temperature (**a**) Relative error analysis of desorption quantity (**b**) Absolute error analysis of coal temperature.

In order to ensure the reliability of the test results, the test instrumentations were calibrated before the experiments. However, the temperature of coal and desorption quantity of methane change with time during methane desorption process, and their truth-values are hardly obtained, so the average value of multiple test results is taken as its truth-value, error analysis is carried on for the test results. Herein, under environment temperature of 283.15 K and initial methane pressure of 1.5 MPa, the desorption tests were repeated three times according to the specification, and the test results and error analysis are showed in Fig. 4.

From Fig. 4, it can be seen that for the three test results of desorption quantity and temperature, both variation tendency and quantitative values are very consistent. Compared with the average values, the relative errors of the adsorption quantity are less than 5%, and the absolute errors of temperature are less than 0.3 K, furthermore mostly of the absolute errors are less than 0.1 K, which fully shows that not only the tests are repeatable, but also the test results are reliable and accurate.

### Evolution of coal temperature during methane desorption inside the canister

**Figure 5:**
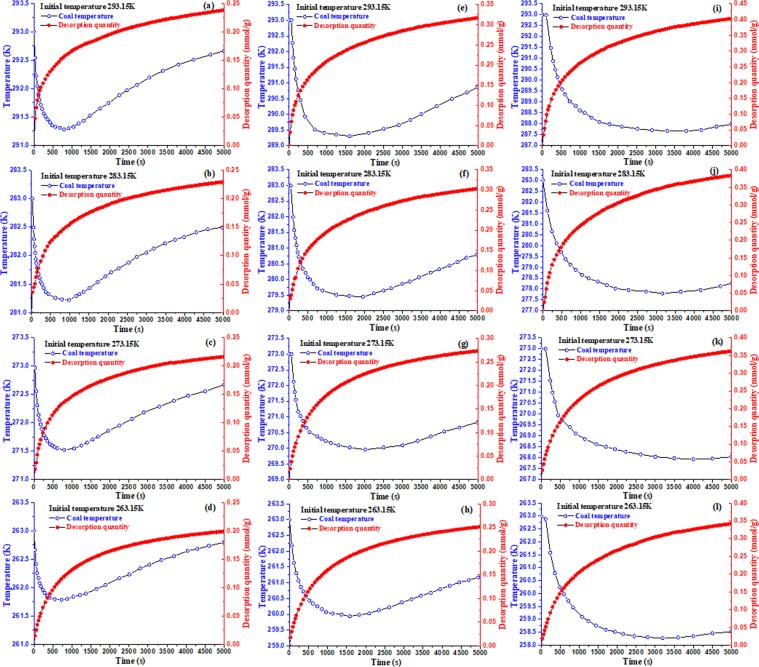
Temperature and desorption quantity variation during methane desorption process in coal at 0.74 MPa (**a–d**), 1.5 MPa (**e–h**) and 2.5 MPa (**i–l**).

To avoid the effect of different initial temperatures and pressures, desorption tests will be conducted under four different initial temperatures (293.15 K, 283.15 K, 273.15 K, 263.15 K) and three different pressure (0.74 MPa, 1.5 MPa, 2.5 MPa). And the results are shown in Fig. 5, the coal temperature evolutions show a similar trend under different temperatures and initial pressure, so do the evolutions of methane desorption quantity. Methane desorption in coal is an endothermic process: in the initial desorption stage, the temperature of the coal sample center decreases rapidly, reaches the nadir value, increases gradually, and finally approximates to the environmental temperature. The desorption quantity of methane increases rapidly in the initial stage and gradually reaches a constant value later.

**Figure 6:**
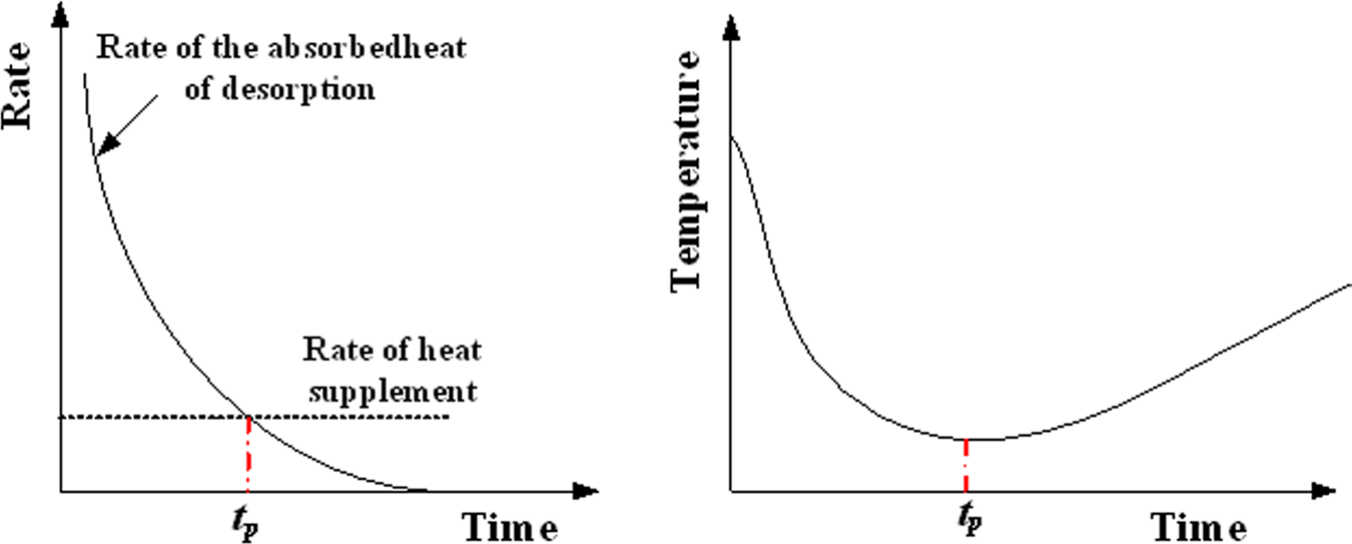
The heat exchange between the absorbedheat of methane desorption and the heat supplement from outside canister.

All of these phenomena are reasonable. For an isobaric desorption process (1 atm), the desorption quantity of methane continuously increases because the adsorbed gas is desorbed from the surface of coal due to differential pressure between the inside and outside of the canister. During the desorption process, the differential pressure becomes smaller and smaller, and the rate of methane desorption decreases rapidly until it almost reduces to be zero. This also means that the absorbedheat of desorption increases during the methane desorption process, which is related to methane desorption quantity. Because the rate of the absorbedheat decreases during the methane desoption process and the rate of heat supplement from the outside of canister remains constant (Fig. 6). The change of coal temperature is caused by the absorbedheat of methane desorption and external heat supplement, in the initial stage of methane desorption, the temperature of coal body will be rapidly reduced to the minimum value, because the absorbedheat of gas desorption is dominant, which is larger than the rate of heat supplement. When the rate of the absorbedheat is equal to the rate of heat supplement, the temperature of coal body begins to slowly rise until the ambient temperature. The rate of heat supplement is related to the position of the coal sample, where the distance is closer to the wall of coal sample canister, the change of temperature of coal sample is less significant, because the heat exchange is larger between the absorbedheat of methane desorption and heat supplement from outside canister.

### Isosteric heats of adsorption on methane desorption

#### Isothermal desorption

**Figure 7:**
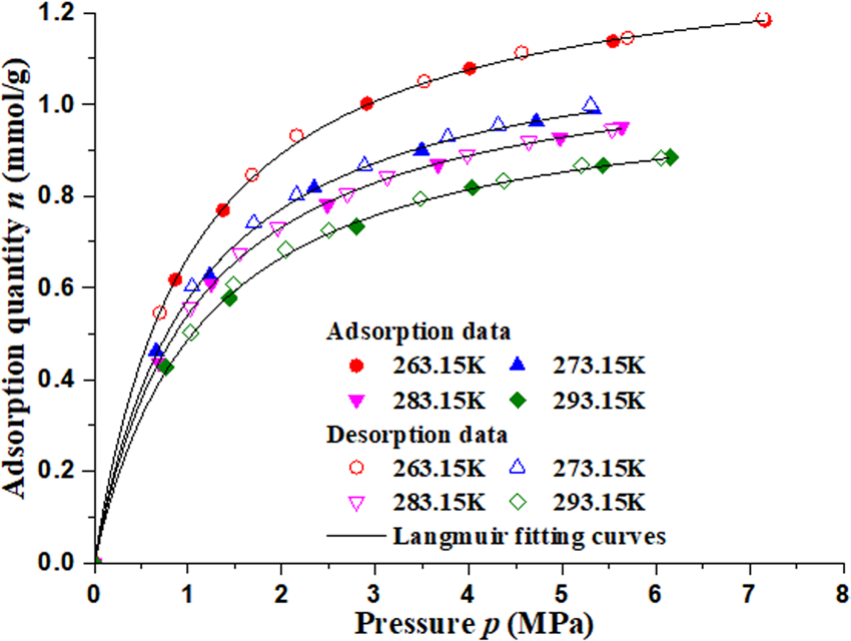
Isothermal adsorption (solid symbols), isothermal desorption (hollow symbols) and langmuir fitting curves of briquette.

Opposite to the isothermal adsorption process, the isothermal desorption is a process of depressurization adsorption, its test process can be described as follows at a set temperature: first, a certain amount of methane in the reference gas cell is filled into the canister. When the pressure in the canister doesn’t change any more for 2 h, the pressure value is recorded. Then, a part of the methane is released from the canister to other vessel (1 atm inside), and when the pressure in the canister is re-stability, the new pressure value is also recorded. So repeatedly, the methane isothermal desorption test results can be obtained at different pressure and so do the other temperatures. The methane isothermal adsorption and desorption data in coal are shown in Fig. 7, and with the increase of temperature, the adsorption quantity of methane decreased.

The isothermal desorption in this coal sample is consistent with isothermal adsorption, which is a reversible process, and they both can be fitted by langmuir equation. The fitting parameters are shown in Table 1, and the correlation coefficients are all above 0.99.

1 $$n=\frac{abp}{1+bp}$$

where *n* is the adsorption quantity of methane under equilibrium temperature (*T*) and pressure (*p*), mmol/g; *a* and *b* are the adsorption constant with unit mmol/g and MPa^−1^ respectively.

**Table 1:** Fitting parameters of isothermal desorption.

Temperature (K)	*a*	*b*	*R*^2^
263.15	1.35	0.97	0.9999
273.15	1.18	0.95	0.9996
283.15	1.13	0.92	0.9999
293.15	1.05	0.87	0.9996

#### Isosteric heats of adsorption

Isosteric heats of adsorption (*q*_*st*_) on methane desorption also can be calculated by Clausius-Clapeyron equation^15^:

2 $$\frac{d\,\mathrm{ln}\,p}{dT}=\frac{{q}_{st}}{R{T}^{2}}$$

where *p* is methane pressure, MPa; *T* is the absolute temperature, K; *q*_*st*_ is the isosteric heats of adsorption, J/mol; *R* is gas constant, herein *R* = 8.314 J/(mol.K).

The integral of Eq. (2) is obtained as follows,

3 $$\mathrm{ln}\,p=-\,\frac{{q}_{st}}{RT}+C$$

where, *C* is the integral constant.

**Figure 8:**
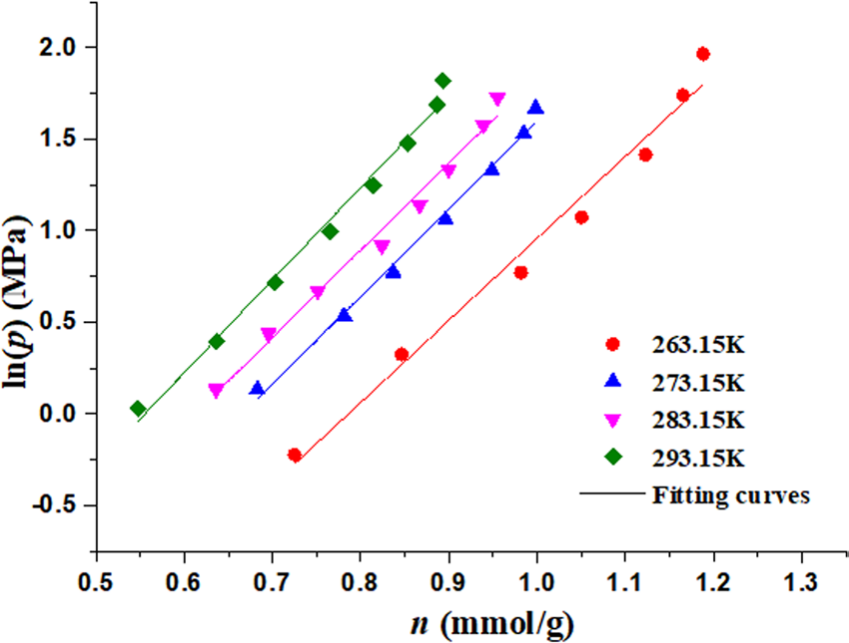
Relationship between pressures(logarithms) and adsorption quantity in desorption process.

From Fig. 7, the corresponding relation of the logarithms of pressure (ln*p*) and adsorption quantity (*n*) can be obtained, and the data are fitted with linear function shown in Fig. 8. The fitting parameters are shown in Table 2.

**Table 2:** Fitting parameters of ln*p*~*n*.

Temperature (K)	ln*p* = *A* + *Bn*		
	*A*	*B*	*R*^2^
263.15	−3.52163	4.4801	0.9781
273.15	−3.19673	4.79681	0.9911
283.15	−2.91173	4.75621	0.9859
293.15	−2.80442	5.05044	0.9887

**Figure 9:**
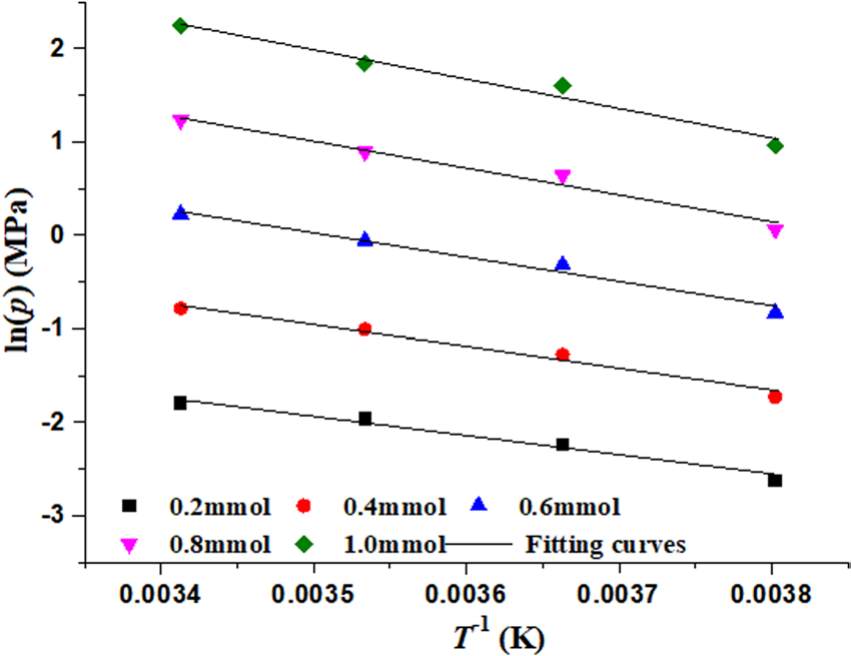
Isosteric lines of desorption process.

The values (*n*) of adsorption quantity are selected as 0.2, 0.3, 0.4, 0.5, 0.6, 0.7, 0.8, 0.9, 1.0 mmol respectively, and according fitting parameters in Table 2, the values (ln*p*) are calculated respectively under 263.15 K, 273.15 K, 283.15 K and 293.15 K respectively. Then corresponding relation of the temperature (*T*^−1^) and the logarithms of pressure (ln*p*) can be obtained and shown in Fig. 9, which satisfy linear relation and the fitting parameters are shown in Table 3.

**Table 3:** Fitting parameters of ln*p*~1*/T*.

*n* (mmol)	ln*p* = *D* + *E/T*		
	*D*	*E*	*R*^2^
0.2	5.25277	−2054.34	0.9835
0.4	7.19314	−2328.33	0.9865
0.6	9.1335	−2602.32	0.9866
0.8	11.0738	−2876.32	0.9851
1.0	13.0142	−3150.31	0.9829

**Figure 10:**
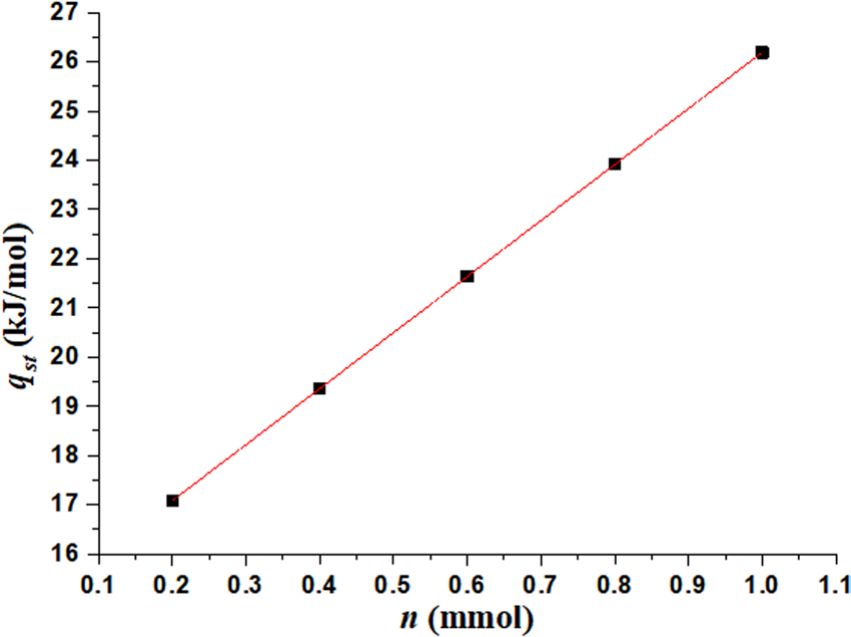
Isosteric heats of adsorption with adsorption quantity.

According to Eq. (3) and Table 3, Isosteric heats of adsorption (*q*_*st*_) can be calculated by relation formula (*q*_*st*_ = −*RE*) and shown in Fig. 10. It can be seen from Fig. 10 that isosteric heats of adsorption (*q*_*st*_) is linear correlation with adsorption quantity (*n*) and it fitting formula is expressed as,

4 $${q}_{st}=14.8017+11.3898n$$

### Desorption quantity of methane

**Figure 11:**
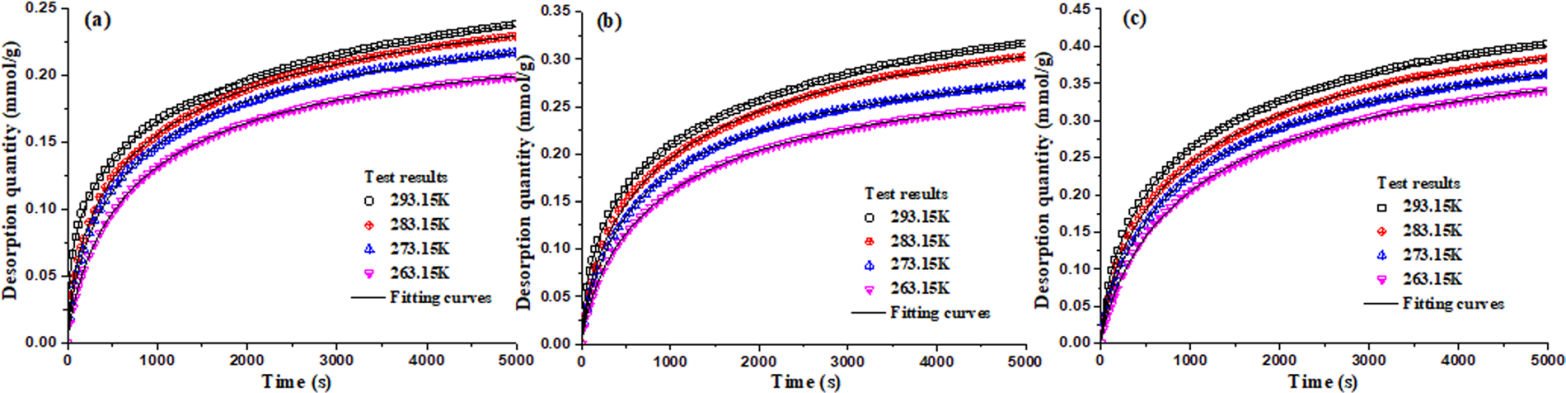
Desorption quantity of methane with time under different equilibrium temperatures and initial pressures (**a)** 0.74 MPa (**b**) 1.5 MPa (**c**) 2.5 MPa.

The isothermal desorption of methane in coal were conducted under different temperatures (263.15 K, 273.15 K, 283.15 K and 293.15 K) and pressures (0.74 MPa, 1.5 MPa and 2.5 MPa), and the results of desorption quantity with time are shown in Fig. 11. It can be seen from Fig. 11 that the higher the environmental temperature is, the faster the methane desorbs. The desorption quantity of methane is fitted by Eq. (5)^16^, and the fitting parameters are shown in Table 4.

5 $${n}_{d}=\frac{{n}_{\max }\alpha {t}^{\gamma }}{1+\alpha {t}^{\gamma }}$$

where *n*_*d*_ is the cumulative desorption quantity of methane, mmol/g; *n*_*max*_ is the maximum desorption quantity, mmol/g; *α* and *γ* are the fitting parameters; *t* is the desorption time, s.

**Table 4:** Fitting parameters of methane desorption process.

Pressure/Temperature		293.15 K	283.15 K	273.15 K	263.15 K
0.74 MPa	*n*_max_	0.42743	0.31391	0.27593	0.24670
	*α*	0.03293	0.01278	0.00781	0.00431
	*γ*	0.42742	0.62855	0.72163	0.80700
	*R*^2^	0.9996	0.9996	0.9993	0.9999
1.5 MPa	*n*_max_	0.47139	0.40778	0.34758	0.32080
	*α*	0.01397	0.00691	0.00522	0.00389
	*γ*	0.58601	0.70830	0.77090	0.80300
	*R*^2^	0.9996	0.9994	0.9999	0.9999
2.5 MPa	*n*_max_	0.55316	0.51880	0.47918	0.45001
	*α*	0.00845	0.00573	0.00412	0.00273
	*γ*	0.67651	0.72869	0.77804	0.82826
	*R*^2^	0.9994	0.9992	0.9999	0.9997

During the methane desorption process in coal, the cumulative desorption quantity (*n*_*d*_) of methane at any time can be expressed with initial adsorption quantity (*n*_*a*_) and methane adsorption quantity (*n*) at this moment.

6 $${n}_{d}={n}_{a}-n$$

Substituting Eq. (6) into Eq. (4), Isosteric heats of adsorption (*q*_*st*_) can be given as follows,

7 $${q}_{st}=14.8017+11.3898\,({n}_{a}-{n}_{d})$$

### Evolution of the total specific power during methane desorption process in coal

If *q*_*st*_ is defined as the endothermic power induced by the heat of methane desorption in coal, if per mole of gas is desorbed from per unit mass of coal, the total specific power (TSP) *W*(*t*) can be expressed as Eq. (8)^13^.

8 $$W(t)={q}_{st}\frac{d{n}_{d}}{dt}$$

where *dn*/*dt* is the rate of methane desorption, mmol/(g.s). It can be gotten by taking the time derivative of both sides of Eq. (5),

9 $$\frac{d{n}_{d}}{dt}=\frac{{n}_{\max }\alpha \gamma {t}^{\gamma -1}}{{(1+\alpha {t}^{\gamma })}^{2}}$$

Substituting Eq. (7) and Eq. (9) into Eq. (8), the TSP of *W*(*t*) is obtained,

10 $$W(t)=[14.8017+11.3898({n}_{a}-\frac{{n}_{\max }\alpha {t}^{\gamma }}{1+\alpha {t}^{\gamma }})]\frac{{n}_{\max }\alpha \gamma {t}^{\gamma -1}}{{(1+\alpha {t}^{\gamma })}^{2}}$$

**Figure 12:**
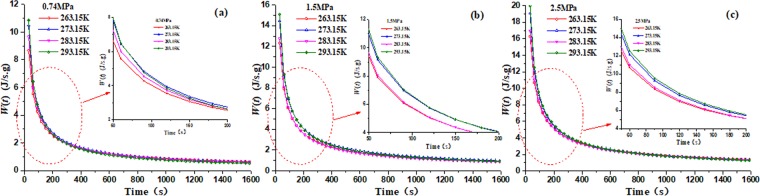
The TSP of *W*(*t*) in the process of methane desorption in coal (**a**) 0.74 MPa (**b**) 1.5 MPa (**c**) 2.5 MPa.

Figure 12 shows that the change laws of the total specific power during methane desorption, which are obtained by substituting the parameters of Table 1 and Table 4 into Eq. (1) and Eq. (10). It can be seen from Fig. 12 that in the initial stage of methane desorption the TSP of *W*(*t*) decreases sharply and then decreases slowly, which is obvious that TSP of *W*(*t*) is directly related to the methane desorption rate, and the slower the methane desorption, the smaller the total specific heat power, when methane no longer desorbs from coal, the total specific heat power tends to zero. Moreover, it is easy to find out that the differences of total specific heat power are not obvious at different temperatures with the same adsorption equilibrium pressure. However, the total specific power is larger under higher initial equilibrium pressure than that under lower initial equilibrium pressure.

## Heat Exchange Model in Coal During Gas Desorption

### Establishment of heat exchange model

**Figure 13:**
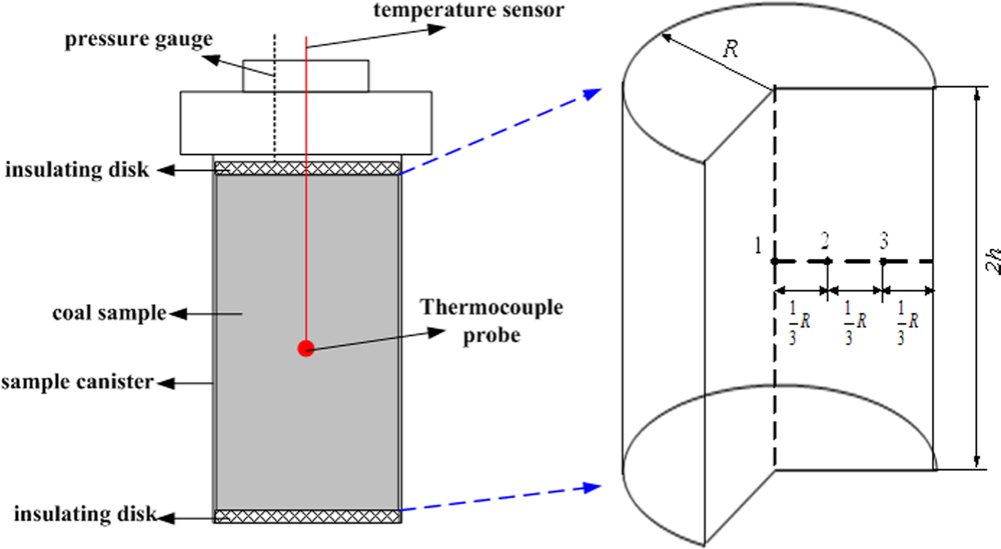
Simplified heat exchange model of coal column.

Methane desorption is a complex thermal-dynamic process of heat and mass transfer. When methane molecules separate themselves from different adsorption sites on coal surface, the differences of energy obtained from coal body vary greatly, which is due to the complex porous structure in coal. These characteristics make it difficult to describe the thermal-dynamic process of methane desorption. And it is also difficult to test the temperature at more positions in the existing experimental equipment, herein, only coal center temperature is tested in this paper which is limited by the size and tightness of the device. Therefore, in order to reveal the distribution law of coal temperature inside the canister during methane desorption process, it is necessary to establish a reasonable and effective heat transfer model, which is consistent with the test as shown in Fig. 13. Considering the complexity of this physical model, the following assumptions are made here: (1) The thermal properties of coal are replaced by equivalent parameters, which are isotropic; (2) The effect of methane desorption in unit volume coal is the same; and (3) the absorbedheat is constant for desorbing equal amount of methane.

Based on above assumptions, the change of coal temperature at any position can be calculated as the dynamic heat exchange problem with an unsteady inner thermal source with the third boundary, and the polar equation can be expressed as,

11 $$\frac{\partial T}{\partial t}=\alpha (\frac{{\partial }^{2}T}{\partial {r}^{2}}+\frac{1}{r}\frac{\partial T}{\partial r}+\frac{{\partial }^{2}T}{\partial {z}^{2}})-\frac{W(t)}{c}\,(0 < r < R,\,0 < z < h)$$

Initial condition: *T*|_*t*=0_ = *T*_0_

$$\begin{array}{cc}{\rm{Boundary}}\,{\rm{conditions}}: & \frac{\partial T}{\partial r}+\frac{\alpha }{\lambda }(T-{T}_{0})=0\,(r=R,\,t > 0)\\  & \frac{\partial T}{\partial z}+\frac{\alpha }{\lambda }(T-{T}_{0})=0\,(z=\pm h,\,t > 0)\end{array}$$

Where, *T* is the coal temperature; *t* is time; *z* is the central axis of coal column, and its origin is at the center of coal-body; *c* is the specific heat capacity of coal; *α* is the temperature diffusivity of the coal, *α* = *λ*/*ρc*, *λ* and *ρ* are the thermal conductivity and the density of coal; *R* is the radius of coal column inside the canister; *h* is the half height of coal column; *T*_0_ is not only initial temperature of coal-body but also ambient temperature.

In any longitudinal section of coal column inside the canister, as shown in Fig. 13, the temperature at any position along the radial direction can be calculated at any time *t* with the finite difference method^17,18^. For example, the temperatures at positions 3, 2, 1 will be given to reveal the heat exchange between the absorbedheat of methane desorption and the heat supplement *via* the canister along the radial direction with external environment.

### Calculation results and analysis of desorption evolution

The sizes of coal column in the model are same to the test, the radius (*R*) is 32.6 mm and the height (2 *h*) is 100.66 mm. The density of coal (*ρ*) is 1380.35 kg/m^3^. The specific heat capacity (*c*) and thermal conductivity (*λ*) are related to the coal temperature, which can be obtained by the experiments and expressed as following fitting equations^13^,

12 $$c=10.564T-2378.56$$

13 $$\lambda =0.00128T-0.22336$$

According to the corresponding temperature of coal body, including the initial temperature, the specific heat capacity (*c*) and thermal conductivity (*λ*) can be obtained easily by Eq. (12) and Eq. (13). Combined with Eq. (10)~Eq. (13), the heat exchange can be calculated under different initial gas equilibrium pressures and initial temperatures

**Figure 14:**
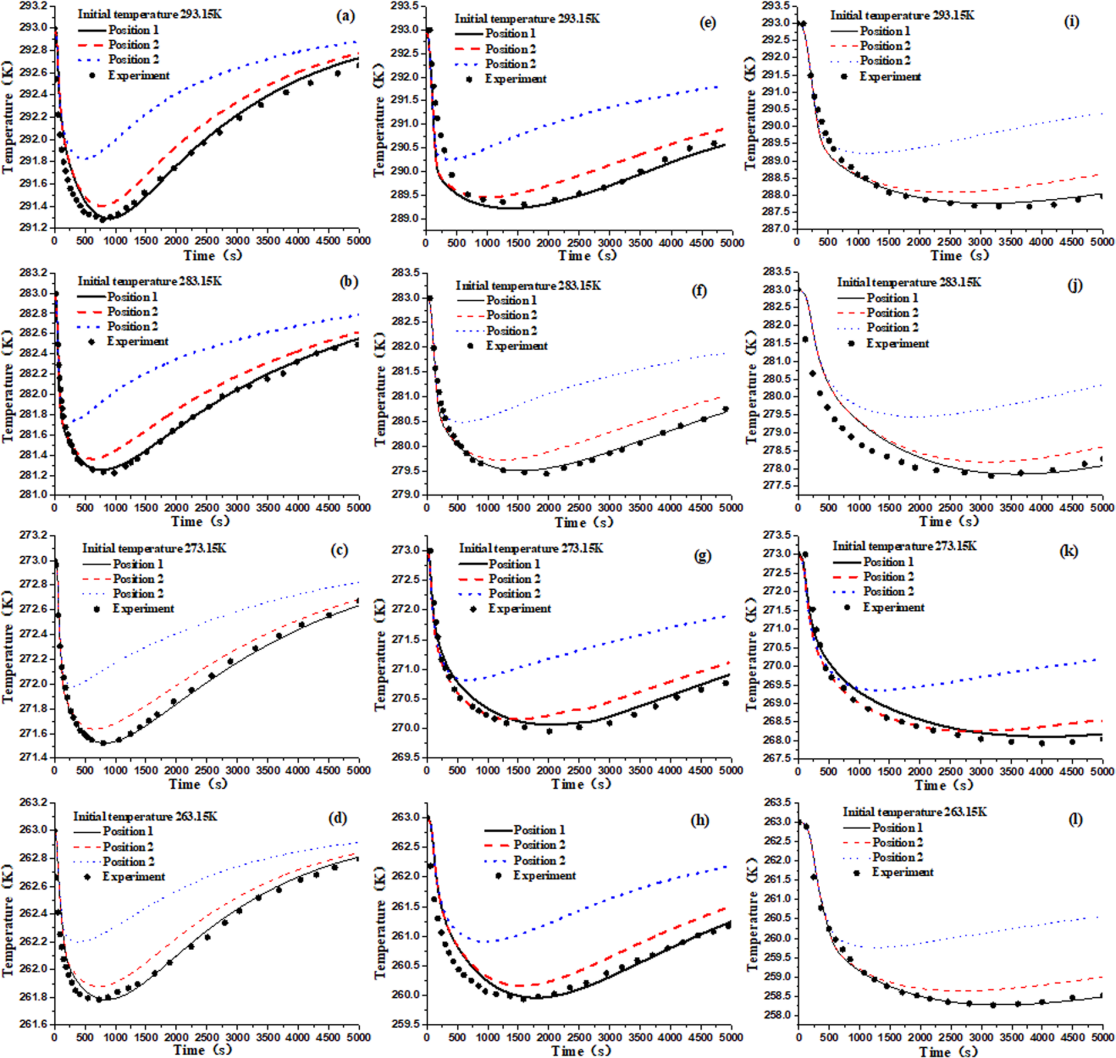
Temperature evolution in coal during methane desorption process at 0.74 MPa (**a–d**), 1.5 MPa (**e–h**) and 2.5 MPa (**i–l**).

**Figure 15:**
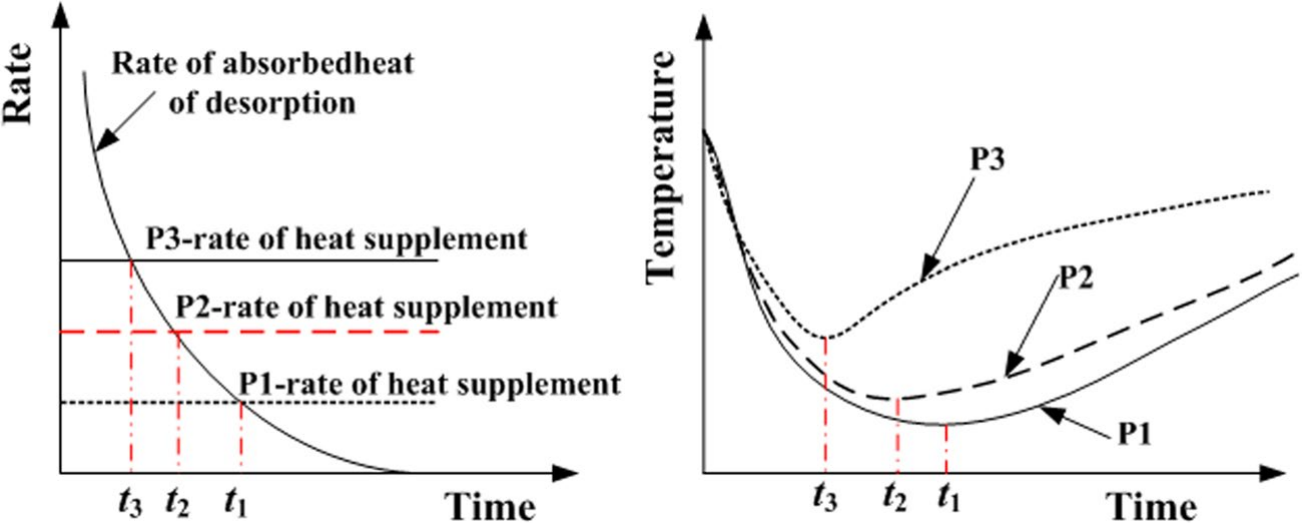
Temperature evolution at three positions in coal body.

The modeling results show the similar tendency with the test results at different positions in coal body, which decrease sharply to an extremely low value, then slowly rise to the previous ambient temperature (Fig. 14). And the closer away from the wall of the coal sample canister, the smaller the decrease of the coal body temperature is, and the shorter the time is to reach extremely low value, which are shown in Fig. 13 of the following positions 3, 2, 1. The closer to the wall of coal sample canister is, the higher the heat transfer efficiency is, the smaller the change of extreme temperature is, and the shorter time is needed to reach the extreme value. This phenomenon is attributed to the differences of heat transfer efficiency at different positions in coal (Fig. 15).

It can be seen from Fig. 14 that the reduction of coal temperature at extremely low value in the coal center increases gradually with increasing the initial equilibrium pressure under the same set temperature, e.g. at 273.15 K, the maximum reduction of coal temperature are 1.48 K, 3.04 K and 5.07 K respectively under initial equilibrium pressure 0.74 MPa, 1.5 MPa and 2.5 MPa; and at 293.15 K the maximum reduction of coal temperature are 1.72 K, 3.8 K and 5.33 K respectively under initial equilibrium pressure 0.74 MPa, 1.5 MPa and 2.5 MPa, which is consistent with the test results (3~5 K) in danger area of coal and gas outburst. However, the absorbedheat of desorption is exchanged sufficiently with the heat supplement of external environment, and the time is sufficiently long, the coal temperature will tend to be ambient temperature. In this test, at same environmental temperature, with the initial pressure increasing, the time becomes longer for the entire coal sample temperature to rise up to the previous temperature. This does indicates that the absorbedheat of desorption is really prominent and nonnegligible in the coal seam, which is likely to be the precursor of coal and gas outburst. If the monitoring method is used to detect the temperature of coal seam in real time, and once the temperature of coal body drops sharply, the necessary safety measures should be taken in time.

## Conclusions

This paper proves the temperature reduction of coal body induced by the absorbedheat in the process of methane desorption. The coal temperatures are tested under four different environment temperatures (263.15 K, 273.15 K, 283.15 K and 293.15 K) and three different pressures (0.74 MPa, 1.5 MPa, 2.5 MPa), and the thermal-dynamic model of methane desorption is developed. The test results and numerical simulation results will well reveal the mechanism of coal temperature evolution during the methane desorption process, which can be as an index for predicting coal and gas outburst. Then several conclusions can be obtained as following:

1. The isothermal desorption is a process of depressurization adsorption, which is a reversible process of isothermal adsorption. And isosteric heats of adsorption for desorption process is linear correlation with adsorption quantity.
2. The coal center temperature goes down by 5.5 K with the heat transfer of the absorbedheat and the heat supplement from external environment in the process of methane desorption, which indicates that the anomalous temperature is obvious in coal and may cause coal and gas outburst.
3. The methane desorption evolution shows a similar tendency with time under different environment temperatures, which can be expressed by exponent function. The temperature of coal sample at any position decrease sharply to a minimum value, then begins to slowly rise until the ambient temperature.
4. The TSP of *W*(*t*) is directly related to the methane desorption rate, which is decreases exponentially. And as a index, TSP is used to describe the thermal-dynamic process at any position in coal body. And the closer to the wall of coal sample canister, the smaller the change of extreme temperature, and the shorter time is needed to reach the extreme value.
5. With the initial equilibrium pressure increasing, the extreme value of coal temperature increases gradually under same environmental temperature, and it will take much longer time to go up to its original temperature.
